# Dynamic tuning of terahertz atomic lattice vibration via cross-scale mode coupling to nanomechanical resonance in WSe_2_ membranes

**DOI:** 10.1038/s41378-024-00827-w

**Published:** 2025-01-22

**Authors:** Bo Xu, Zejuan Zhang, Jiaze Qin, Jiaqi Wu, Luming Wang, Jiankai Zhu, Chenyin Jiao, Wanli Zhang, Juan Xia, Zenghui Wang

**Affiliations:** 1 Institute of Fundamental and Frontier Sciences, University of Electronic Science and Technology of China, Chengdu, 610054 China; 2 Hubei Key Laboratory of Micro-Nanoelectronic Materials and Devices, Hubei University, Wuhan, 430062 China; 3 State Key Laboratory of Precision Measuring Technology and Instruments (Tianjin University), Tianjin, 300350 China; 4 School of Integrated Sciences and Engineering (Exemplary School of Microelectronics), University of Electronic Science and Technology of China, Chengdu, 610054 China; 5 State Key Laboratory of Electronic Thin Films and Integrated Devices, University of Electronic Science and Technology of China, Chengdu, 610054 China

**Keywords:** NEMS, Nanoscale materials

## Abstract

Nanoelectromechanical systems (NEMS) based on atomically-thin tungsten diselenide (WSe_2_), benefiting from the excellent material properties and the mechanical degree of freedom, offer an ideal platform for studying and exploiting dynamic strain engineering and cross-scale vibration coupling in two-dimensional (2D) crystals. However, such opportunity has remained largely unexplored for WSe_2_ NEMS, impeding exploration of exquisite physical processes and realization of novel device functions. Here, we demonstrate dynamic coupling between atomic lattice vibration and nanomechanical resonances in few-layer WSe_2_ NEMS. Using a custom-built setup capable of simultaneously detecting Raman and motional signals, we accomplish cross-scale mode coupling between the THz crystal phonon and MHz structural vibration, achieving GHz frequency tuning in the atomic lattice modes with a dynamic gauge factor of 61.9, the best among all 2D crystals reported to date. Our findings show that such 2D NEMS offer great promises for exploring cross-scale physics in atomically-thin semiconductors.

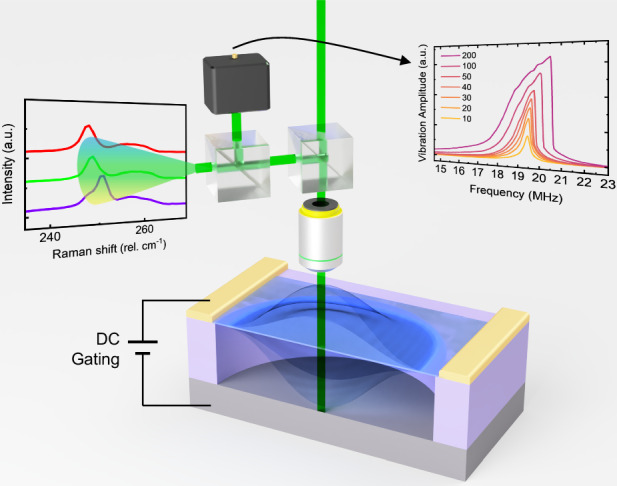

## Introduction

Two-dimensional (2D) semiconductors have garnered significant interests in materials science and electronics research, offering new opportunities for high-performance devices and circuits beyond Moore’s law^1–3^. Among these materials, tungsten diselenide (WSe_2_) stands out with its superior transistor performance and unique ability to control polarity without doping^4–9^. When further combined with its mechanical degree of freedom, WSe_2_-based nanoelectromechanical systems (NEMS) exhibit intriguing qualities such as high quality factor^10^ and superb electrostatic frequency tuning efficiency^11^, highly promising for fundamental research such as investigating exciton-optomechanical coupling^12^ and applications such as signal processing, sensing^13^, and computation^14^.

Particularly, with their atomically-thin vibrational structure, such NEMS resonators can easily generate substantial tensile stress and out-of-plane displacement even at sub-nanometer scales, making them an ideal platform for exploring dynamic strain engineering and cross-scale vibration coupling in 2D materials^15–18^. Such potential is further endorsed by the encouraging success in exploring both THz atomic lattice vibration^19,20^ and MHz nanomechanical vibrations in 2D WSe_2_^10,11^, enabling the study of dynamic tuning of quasiparticles (excitons or phonons)^21^ through collective mechanical vibration.

However, this unique possibility still remains largely unexplored for WSe_2_ NEMS, representing a major lost opportunity for harnessing the mechanical degree of freedom across multiple frequency bands in this 2D semiconductor. Further, this impedes the potential realization of WSe_2_ NEMS-based frequency modulators in which the MHz electrical excitation could be leveraged to effectively tune the THz atomic lattice vibration, and hinders the exploration of many other innovative device applications.

**Fig. 1:**
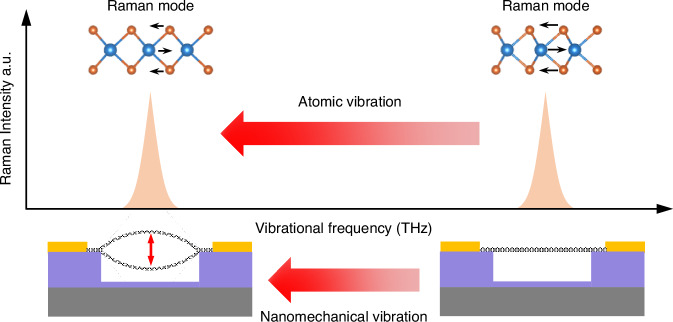
Illustration of atomic lattice and nanomechanical vibration coupling for WSe_2_ samples. The schematics show atomic lattice vibration mode of WSe_2_ crystal (top) modulated by device resonance (bottom)

In this work, we demonstrate cross-scale dynamic coupling between atomic lattice vibration and nanomechanical resonance in few-layer WSe_2_ NEMS (Fig. 1). Utilizing both frequency and amplitude degrees of freedom in vibration actuation, we finely control and effectively amplify the dynamic motion in the WSe_2_ membrane, from 19.4 to 21.7 MHz and from 0.7 nm to 19.9 nm, resulting in a maximum strain of 130 ppm. Leveraging both elastic and inelastic light scattering processes, we use just a single detection laser and simultaneously capture both nanomechanical and Raman vibrational signals, overcoming their orders-of-magnitude of frequency difference (MHz vs. THz). This allows successful observation of a 61.89 GHz (~2.1 cm^−1^) softening in the 7.68 THz (256.1 cm^−1^) Raman mode, corresponding to a dynamic gauge factor of 61.9, the best among all 2D crystals reported to date. Our results demonstrate that these 2D resonators enable the unique capability of manipulating THz vibrational modes through precise tuning of MHz nanomechanical resonances, offering great promises for exploring and exploiting cross-scale physical processes in these atomically-thin semiconductors.

## Results and discussion

We fabricate 2D WSe_2_ NEMS resonators using a dry transfer technique (details shown in “Methods” section)^11,22,23^. In order to achieve a smooth strain distribution, we choose fully covered circular drumhead as device structure which exhibits highly symmetric vibrational shape in the fundamental mode^24–27^. To accurately determine the device thickness, we employ ultra-low-frequency (ULF) Raman, high frequency Raman, and photoluminescence (PL) measurements^28–30^ (Fig. S1). Among the optical signatures, in principle the high frequency Raman modes manifest the lattice vibration, and thus should be sensitive to deformation in the crystal lattice (top insets in Fig. 1). We therefore focus on studying the dynamic tuning of these THz modes in the crystal, using the vibrational strain generated through the MHz modes in the NEMS resonators. This further enables exploration of the cross-scale coupling between the atomic lattice vibration and nanomechanical vibration.

**Fig. 2:**
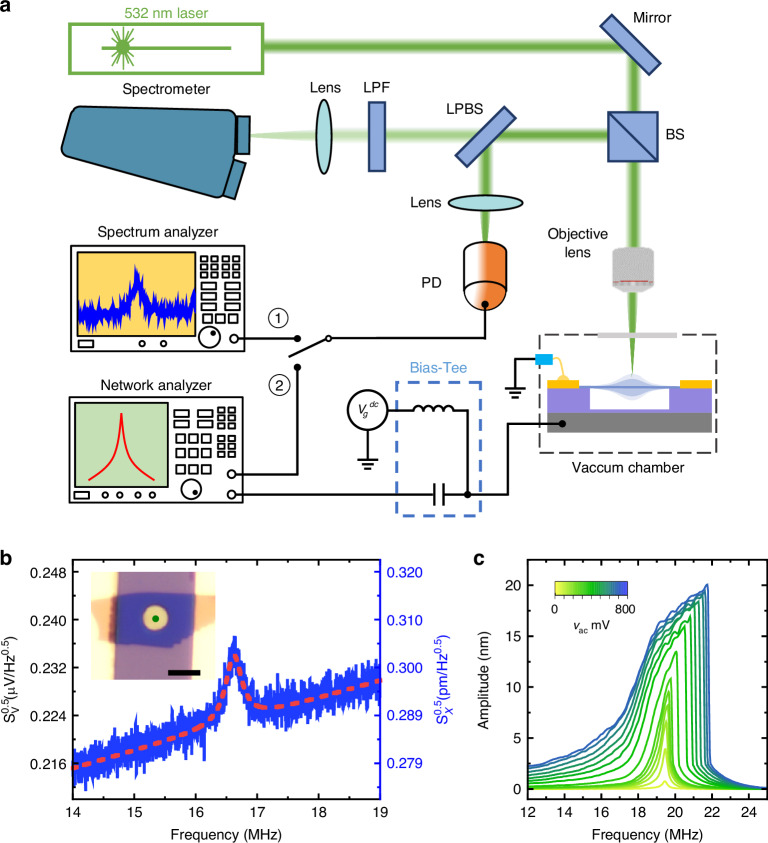
Experimental measurements with nanomechanical vibrations. **a** Schematic illustration of the measurement system. **b** Thermomechanical (un-driven) resonances measured from a 4 L circular drumhead WSe_2_ NEMS resonator. The optical image of the 4 L WSe_2_ NEMS resonator with diameter *d* = 4 μm (scale bar: 5 μm) is shown in the inset, in which the green dot indicates the laser spot position. **c** Driven resonances measured at *V*_g_ = 7 V with different AC driving amplitudes (2 mV to 800 mV), showing the transition from linear to nonlinear response for the 4 L WSe_2_ NEMS resonator shown in (**b**)

To simultaneously detect and monitor both the MHz and THz vibrational modes, we devise a custom-built measurement setup which integrates laser interferometry with Raman spectroscopy. We focus on the fundamental resonance mode of the circular device (METHODS), and focus the laser spot at the center where the dynamic strain is highly uniform and vibrational amplitude is the greatest for the fundamental mode. As shown in Fig. 2a, the long-pass-beam-splitter (LPBS) divides the reflected light signal into two paths: the elastically scattered (Rayleigh) light signal, carrying the interferometric MHz motional signal of the NEMS device, is reflected at the LPBS and directed to a photodetector (PD), which is further analyzed using spectrum analyzer or network analyzer. Simultaneously, the inelastically scattered (Raman) photons pass through the LPBS and enter the spectrometer, which are used to monitor the THz phonon modes in the WSe_2_ crystal. All measurements are conducted under vacuum (~10^−3 ^mbar) at room temperature.

Leveraging the electrostatic actuation capability in the custom-built setup, we excite nanomechanical resonance in the devices to generate dynamic strain. We start with measuring the intrinsic resonant response in the absence of electrical driving (thermomechanical noise), with the result in Fig. 2b showing a clear nanomechanical resonance of MHz frequency. To generate significant dynamic strain in the device, we drive the device motion electrostatically near its resonance frequency, by applying a DC + AC voltage between the gate electrode and the 2D membrane^31^. For this capacitor, the dynamic electrostatic driving force is:

1 $${F}_{d}\approx \frac{dC}{dx}{V}_{g}{v}_{ac}$$

which depends on both the DC and AC voltages. Upon exploring the parameter space, we choose to operate this particular device (inset in Fig. 2b) at *V*_g_ = 7 V and *v*_ac_ from 2 mV to 800 mV, for effective motion actuation and signal transduction (details shown in Methods section).

In order to generate sufficient strain, we intensely drive the device beyond its linear dynamic range^32^ such that nonlinear effects must be considered. Here we employ the Duffing equation to describe the nonlinear resonant response^33–35^:

2 $${{{x}}}^{\prime\prime}+{{\beta }}{{x}}^{\prime} +{4{{\pi}^{2}}f}_{0}^{2}x+a{x}^{3}={F}_{d}/m$$

Here, *x* is the out-of-plane vibrational displacement, $2{\pi}f_{0}$ is the angular resonance frequency, *F*_d_ is the driving force applied, *m* is the modal mass, *β* is the dissipation term, and *α* is the cubic nonlinear term. The analytical solution (METHODS) resembles the linear resonance peak at small amplitudes, and becomes a shark-fin shape with increased driving (Fig. S2).

The measured response (Fig. 2c) exhibits shark-fin-like shape, which can be approximated by the Duffing equation (Fig. S2), and shows that the vibrational amplitude in the nonlinear regime can become significantly larger than that in the linear regime. This suggests that the dynamic strain can be very effectively generated by harnessing the nonlinear resonant response. From the measured signal amplitude, we estimate a maximum dynamic strain of 130 ppm in Fig. 2c, much greater than that without driving (Fig. 2b, 0.00288 ppm) (details shown in METHODS). This demonstrates that leveraging the resonant amplification effect^36^ and driving the device into deep nonlinearity, we can achieve 5 orders of enlargement in generating dynamic strain, offering an effective tool for tuning the atomic lattice vibration^37,38^.

**Fig. 3:**
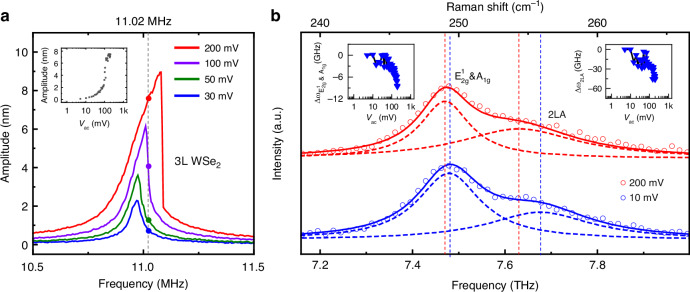
Nanomechanical and Raman vibrations measured from the 3 L WSe_2_ resonator with *d* = 4 μm, using *V*_g_ = 3 V, and *v*_ac_ from 30 mV to 200 mV (Full data with *v*_ac_ from 5 mV to 200 mV are shown in Fig. S10). **a** Select amplitude-frequency curves showing nanomechanical responses under different *v*_ac_. The insets in (**a**) show vibrational amplitude of WSe_2_ membrane as a function of *v*_ac_ at 11.02 MHz. **b** Select Raman spectra with the device driven at driving frequency 11.02 MHz, *V*_g_ = 3 V, showing E_2g_^1^ & A_1g_ and 2LA modes, when *v*_ac_ = 200 mV (red, device in nonlinear regime) and *v*_ac_ = 10 mV (blue, device in linear regime). Symbols are measured spectra data and dashed lines are fittings to the individual peaks, with solid lines being the sums. The insets in (**b**) show Raman peak shift ∆ω_E2g_^1^_& A1g_ (left) and ∆ω_2LA_ (right) as a function of *v*_ac_ at 11.02 MHz (indicated by colored triangles). The measurement conditions in (b) largely correspond to the symbols in (**a**)

We next examine such dynamic tuning of the THz Raman modes using the 11.02 MHz nanomechanical vibration, with the results shown in Fig. 3. Specifically, we measure the Raman response on resonance (dashed line in Fig. 3a) under different driving amplitudes. Three atomic vibrational modes can be identified from the WSe_2_ Raman spectra, namely E_2g_^1^ & A_1g_ and 2LA, and are all sensitive to strain. These modes can be effectively utilized to characterize the dynamic strain in WSe_2_ NEMS resonators. The Raman spectra (Fig. 3b) clearly illustrate that the peak positions of the E_2g_^1^ & A_1g_ and 2LA modes exhibit clear redshift as driving amplitude increases, specifically when the device motion enters the nonlinear regime. The inset of Fig. 3b summarizes the evolution of both E_2g_^1^ & A_1g_ and 2LA modes as the AC driving increases from 5 mV to 200 mV, showing clear and consistent softening behavior with a maximum downshift reaching 1.5 cm^−1^ (45 GHz). Our observation evidences that the dynamic strain induced by the strong nonlinear vibration can lead to clear phonon softening in the 2D crystal^39,40^. Interestingly, this suggests that the MHz nanomechanical resonance can be used to effectively tune the THz phonon modes, resulting in GHz frequency shift in the atomic lattice vibration.

**Fig. 4:**
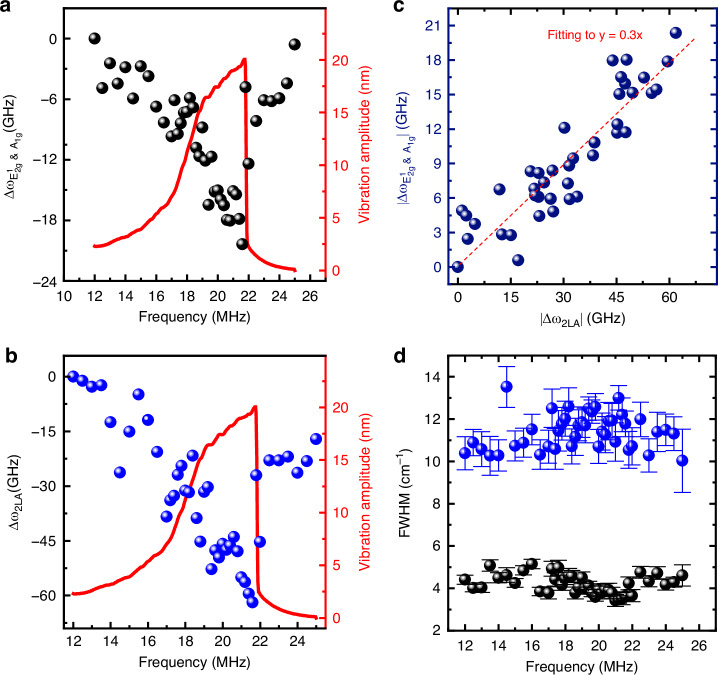
Resonance and Raman spectra measured from the 4 L WSe_2_ (*d* = 4 μm) with *V*_g_ = 7 V and *v*_ac_ = 800 mV. **a**, **b** Measured nonlinear resonance spectrum (red, right axis) and Raman peak shift. **a** ∆ω_E2g_^1^_& A1g_ and (**b**) ∆ω_2LA_ under different driving frequencies (black and blue symbols, left axis) plotted alongside the resonance response curves. **c** The extracted relationship between E_2g_^1^ & A_1g_ peak shift (∆ω_E2g_^1^_& A1g_) and 2LA (∆ω_2LA_). **d** FWHM for E_2g_^1^ & A_1g_ and 2LA Raman peaks under different driving frequencies. Fitting details of Raman spectra at different driving frequencies are shown in Fig. S3

By examining the solution to the Duffing equation (METHODS), we find that the vibrational amplitude depends on not only the driving force but also the excitation frequency, and so does the dynamic strain. This suggests the opportunity of fine tuning the phonon modes in the 2D crystal by manipulating the driving frequency of the NEMS resonator. Specifically, we perform the frequency sweeping measurements when setting DC voltage *V*_g_ = 7 V and AC driving amplitude *v*_ac_ = 800 mV, with the upward sweep results shown in Fig. 4 (downward sweep data in Fig. S6). Interestingly, both the E_2g_^1^ & A_1g_ and 2LA modes exhibit clear redshift patterns following the Duffing curve of the nonlinear response as the frequency increases, and form inverted shark-fin features mirroring that of the NEMS resonator (Fig. 4a, b). By analyzing the vibration amplitudes, we determine the maximum dynamic strain to be 130 ppm at 21.6 MHz (Fig. S7). This results in the largest Raman shift softening of 18 GHz (0.6 cm^−1^) for E_2g_^1^ & A_1g_ and 61.9 GHz (~2.1 cm^−1^) for 2LA modes at the pinnacles of the inverted shark-fins, corresponding to gauge factors of 20.39 and 61.9 for the two modes respectively (details shown in METHODS), representing the highest values reported to date in dynamic strain tuning of 2D crystals^15–17^.

We now compare the Raman peak shifts of the E_2g_^1^ & A_1g_ and 2LA modes using the entire data set, and plot them in Fig. 4c. Notably, we observe a clear linear relationship between the Raman frequency shifts (∆*ω*) of the two modes, with a slope of 0.3 which represents the ratio between their gauge factors. Assuming constant values for gauge factors in this range, the high proportionality suggests that the observed softening in both Raman modes are due to the dynamic strain rather than other effects, which simultaneously tune the two modes. Furthermore, through fitting we find that the full width at half maximum (FWHM) of these Raman peak remain nearly unchanged regardless of the dynamic strain (Fig. 4d), implying that the nanomechanical resonance can precisely tune the spring constants without affecting the dissipation rates in these lattice vibrational modes. It’s important to note that all the above observations are repeatable: we perform similar measurements across multiple devices with different thicknesses (Figs. S4–5 and S8–9), and consistently observe all the effects.

## Conclusion

In summary, through simultaneous Raman and interferometry measurements, we successfully demonstrate cross-scale coupling between the MHz nanomechanical resonance modes and THz lattice vibrational modes, and show dynamic tuning of Raman modes under strongly nonlinear resonance. Our findings demonstrate that both the E_2g_^1^ & A_1g_ and 2LA modes exhibit clear softening in response to the dynamic strain, which can be effectively tuned by modulating the driving amplitude or frequency. This highlights the potential of NEMS resonators as an ideal platform for manipulating the atomic lattice vibrations in 2D crystals. Our results offer new possibilities for THz signal modulation and processing and for dynamically manipulating 2D materials at the atomic scale.

## Methods

### Device fabrication

We start with mechanically exfoliating WSe_2_ flakes onto polydimethylsiloxane (PDMS) stamps mounted on glass slides. The thickness of these samples is determined using optical contrast, ultra-low-frequency (ULF) Raman, and photoluminescence (PL) measurements. The optical spectroscopy measurements are highly effective in identifying few-layer WSe_2_ flakes. Next, the selected WSe_2_ samples are aligned and transferred onto a Si/SiO_2_ substrate with a pre-patterned circular micro-cavity, which is completely covered by the flake to form 2D WSe_2_ resonators. The micro-cavity is fabricated by selectively etching the 290 nm SiO_2_ layer, leaving a 50 nm SiO_2_ layer at the bottom of the cavity. To enable electrical driving in NEMS devices, Au/Cr electrodes are deposited near the cavity which contact with WSe_2_ flake.

### Resonance measurement and frequency tuning

Figure 2a shows measurement setup with which we characterize resonant behaviors of WSe_2_ NEMS resonators. The device motions are electrically excited and interferometrically detected. For electrical excitation, we apply an AC + DC signal generated by a network analyzer and a DC power supply. The electrostatic force is:

3 $${F}_{{\rm{e}}}=\frac{1}{2}\frac{dC}{dx}{({V}_{g}+{v}_{ac})}^{2}\approx \frac{1}{2}\frac{dC}{dx}{{V}_{g}}^{2}+\frac{dC}{dx}{V}_{g}{v}_{ac}$$

Here, *x* is the out-of-plane vibrational displacement and *C* is the capacitance between the WSe_2_ membrane and the gate.

The tuning of resonance frequency through gate voltage can be expressed as^33^:

4 $${f}_{{\rm{res}}}=\frac{1}{2\pi }\sqrt{\frac{\frac{\pi {{\varepsilon }}_{0}^{2}}{8(1-{\nu }^{2}){E}_{{\rm{Y}}}t{\gamma }_{0}^{2}}\frac{{R}^{2}}{{d}^{4}}{V}_{{\rm{g}}}^{4}+4.9{E}_{{\rm{Y}}}t{\gamma }_{0}-\frac{{{\varepsilon }}_{0}\pi {R}^{2}}{3{d}^{3}}{V}_{{\rm{g}}}^{2}}{{M}_{{\rm{eff}}}}}\,$$

where *E*_Y_, *ν*, ${{\varepsilon }}_{0}$, ${\gamma }_{0}$, ${V}_{{\rm{g}}}$, *R*, *d*, *t*, *M*_*eff*_ represent Young’s modulus, Poisson’s ratio, vacuum permittivity, initial strain, gate voltage, the radius of device, vacuum gap depth, thickness and effective mass of resonator. From this equation, we can analyze the resonance frequency shift with the gate voltage, *V*_g_.

The motion is detected using a 532-nm laser through interferometry. During the measurement, we adjust the laser power on the WSe_2_ resonators to avoid laser heating effects which can affect the resonance frequency. We also move the laser spot across different positions on the device to confirm that the fundamental mode is measured.

To examine the effect of static strain, we gradually increase the DC voltage from 0 V to 30 V to increase the static strain while maintaining moderate AC driving. We observe a smooth and continuous evolution of the device frequency without any abrupt changes or jumps (Fig. S11). This confirms the absence of mechanically unstable states such as buckling or wrinkling, and shows that our devices constitute stable nanomechanical platforms for subsequent strain modulations.

To examine the effect of dynamic strain, at each DC bias voltage we gradually increase the AC driving amplitude on the network analyzer. We consistently observe device resonance behavior going from linear to nonlinear regime. We find that the maximum dynamic strain occurs at *V*_g_ = 7 V with *v*_ac_ = 800 mV in our experimental setting.

### Gauge factor estimation

We estimate the gauge factor of the two Raman mode in WSe_2_ by first evaluating the dynamic strain. Based on the fundamental mode shape, we assume that dynamic strain induced by vibration in the circular drumhead resonators is mostly uniform. To estimate the dynamic strain, we therefore compare the membrane areas when at the maximum vibration displacement (*S*_vibration_) and when there is no vibration (*S*_0_). They can be calculated by^17,32^:

5 $${{S}}=2\pi {{\int_{0}^{R}}\left[{\left(\frac{\updelta {\rm{x}}}{\sqrt{0.2695}}\frac{\partial {J}_{0}(2.405r/R)}{\partial r}\right)}^{2}+1\right]}^{0.5}rdr$$

Here, $\delta x$ is the vibrational displacement of membrane, *J*_0_ is 0-order Bessel function of the 1st kind and *R* is the radius of the circular membrane. Using this expression, we estimate the dynamic strain of the membrane as:

6 $${{{\gamma }}}_{{\rm{r}}}=\sqrt{{S}_{vibration}/{S}_{0}}-1$$

Then, the gauge factor of Raman mode can be estimated by:

7 $${{GF}}=\frac{\Delta {{\omega }}/{{\omega }}}{{{{\gamma }}}_{{{r}}}}$$

where ${\omega}$ is Raman frequency and $\Delta{{\omega}}$ is Raman frequency shift.

### Solution to the Duffing equation

The Duffing equation, which is used to describe the most common type of nonlinear resonant response, is shown in Eq. (2). We now express the driving force *F*_d_/*m* as *F*cos($2{\pi}{ft}$), and assume a first order approximation of the solution as *x = A*cos($2{\pi}{ft}$), where *A* does not depend on *t* and $2{\pi}{ft}$ is the same frequency as in the driving term, which is mathematically treated as a variable. We then substitute the approximated solution into Eq. (2) and obtain an equation between *A* and driving force *F*:

8 $${\left[({{4{\pi }^{2}f}}_{0}^{2}-{{4{\pi}^{2}}f}^{2}){{A}}+ \frac{3}{4}\upalpha {A}^{3}\right]}^{2}+{({{\beta }}\times{2{\pi}f}A)}^{2}={F}^{2}$$

Considering that $2{\pi}{ft}$, *α*, *β* and *F* are all constant, we can derive the relation between driving frequency *ω* and vibrational amplitude *A*:

9 $${{4{\pi }^{2}f}}^{2}=\frac{2({{4{\pi }^{2}}f}_{0}^{2}+ \frac{3}{4}\upalpha {A}^{2})-{{{\beta }}}^{2}\pm \sqrt{{{{\beta }}}^{4}-4{{{\beta }}}^{2}({4{{\pi}^{2}}f}_{0}^{2}+ \frac{3}{4}\upalpha {A}^{2})-4\frac{{F}^{2}}{{A}^{2}}}}{2}$$

and this expression can be used to plot the calculated response of a Duffing resonator.

## Supplementary information

Supplemental Material
