# Supplementary material for: Dynamic tuning of terahertz atomic lattice vibration via cross-scale mode coupling to nanomechanical resonance in WSe2 membranes
Source: Microsyst Nanoeng. 2025 Jan 22;11:18. doi: 10.1038/s41378-024-00827-w (PMC11754608; doi:10.1038/s41378-024-00827-w)
Supplement: Supplementary file 1 — Supplemental Material [file 41378_2024_827_MOESM1_ESM.pdf]

Supporting Information for

Dynamic tuning of terahertz atomic lattice vibration via cross-scale mode coupling to nanomechanical resonance in WSe_2 membranes

Bo Xu[1,2,3], Zejuan Zhang[1,4], Jiaze Qin[1], Jiaqi Wu[1], Luming Wang[1], Jiankai Zhu[1], Chenyin Jiao[1], Wanli Zhang[4,5], Juan Xia[1], Zenghui Wang[1,5]

1. *Institute of Fundamental and Frontier Sciences, University of Electronic Science and Technology of China, Chengdu 610054, China.*
2. *Hubei Key Laboratory of Micro-Nanoelectronic Materials and Devices, Hubei University, Wuhan 430062, China.*
3. *State Key Laboratory of Precision Measuring Technology and Instruments (Tianjin University), Tianjin 300350, China.*
4. *School of Integrated Sciences and Engineering (Exemplary School of Microelectronics), University of Electronic Science and Technology of China, Chengdu 610054, China.*
5. *State Key Laboratory of Electronic Thin Films and Integrated Devices, University of Electronic Science and Technology of China, Chengdu 610054, China.*

Corresponding Authors: bo_xu@uestc.edu.cn, juanxia@uestc.edu.cn, zenghui.wang@uestc.edu.cn

These authors contributed equally: Bo Xu, Zejuan Zhang

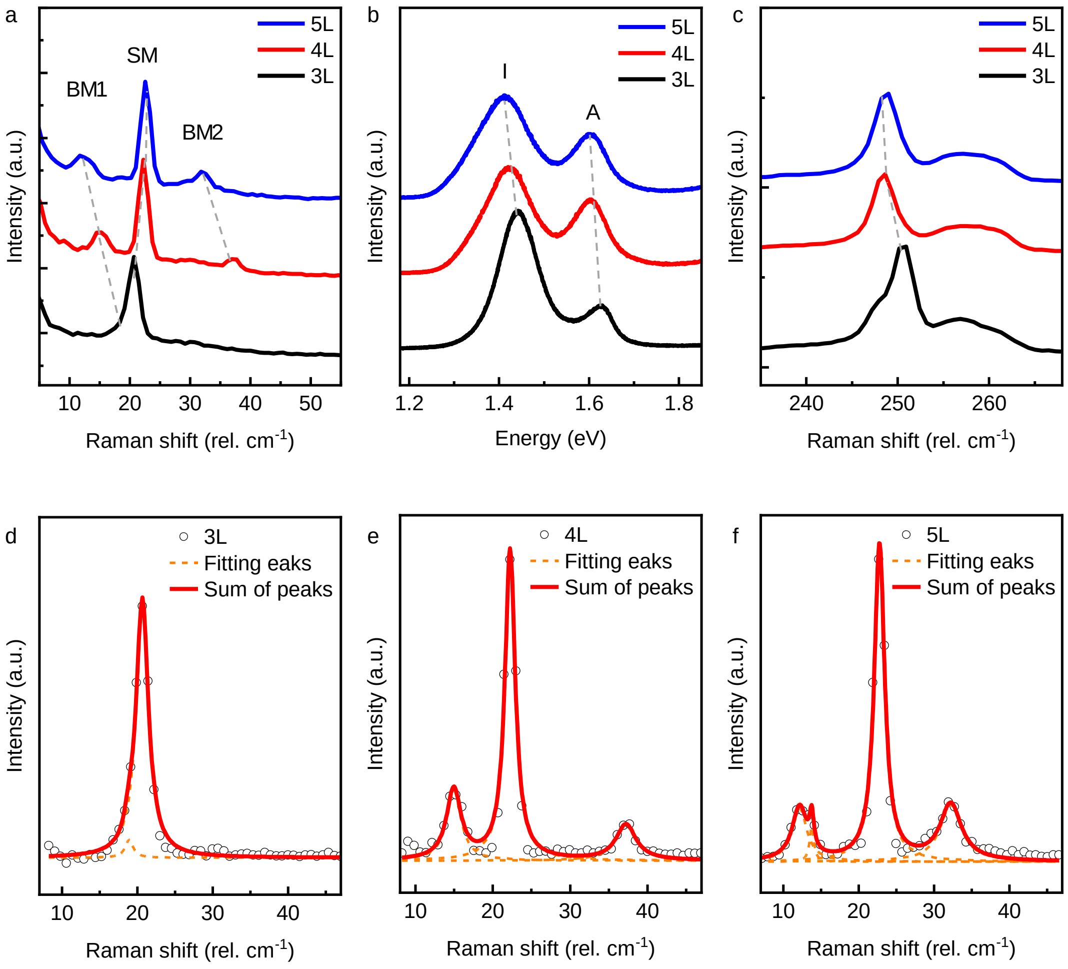

Fig. S1 Spectroscopic characterization of the devices. (a) Thickness-dependent ultra-low frequency (ULF) Raman mode: Shear mode (SM), Breathing mode 1 (BM1) and Breathing mode 2 (BM2), for 3, 4, 5L devices. (b) Photoluminescence (PL) for 3, 4, 5L devices. (c) High-frequency Raman spectra for 3, 4, 5L devices. The grey lines serve as guides to the eyes. (d-f) Fitting of ULF Raman spectra for few-layer WSe_2. The results show that the thicknesses rang from three (3L) to five layers (5L). Specifically, Fig. S1a illustrates the thickness-dependent behavior of the shear mode (SM) and breathing modes (BM1/2) in the Raman spectra. As the thickness decreases from 5L to 3L, the SM frequency decreases from ~22.7 cm^{-1} to ~20.7 cm^{-1}, while the BM1 frequency increases from ~12.1 cm^{-1} to ~18.9 cm^{-1} and BM2 disappears at 3L, as expected. The PL spectra also confirms the number of layers, with both the A exciton and the indirect band gap features show increase in energy with decreasing thickness. Figure S1c shows the thickness dependent high frequency Raman peaks, corresponding to atomic vibrations at around 7.44 THz (248 cm^{-1}) and 7.68 THz (256 cm^{-1}) in few-layer WSe_2, which in principle should be sensitive to deformation in the crystal lattice.

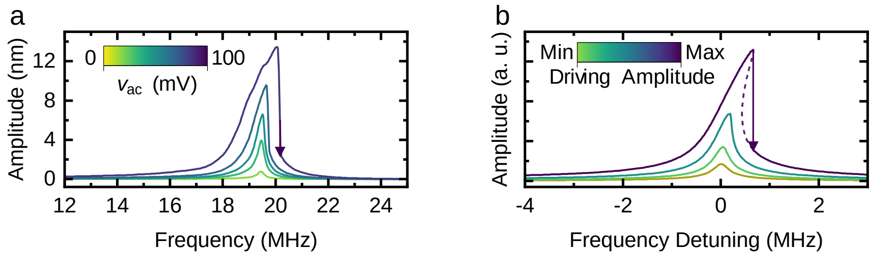

Fig. S2 (a) Resonance response of a 4L device with increasing driving amplitude v_{ac}, showing clear transition from linear to nonlinear regime. The downward arrow indicates the amplitude jump during forward frequency sweep. (b) Calculated frequency response curve (from Eq. 8) of a Duffing resonator with increasing driving amplitude. The arrow indicates the amplitude jump during frequency sweep in practice (such that the dashed curve part cannot be experimentally observed in forward frequency sweep).

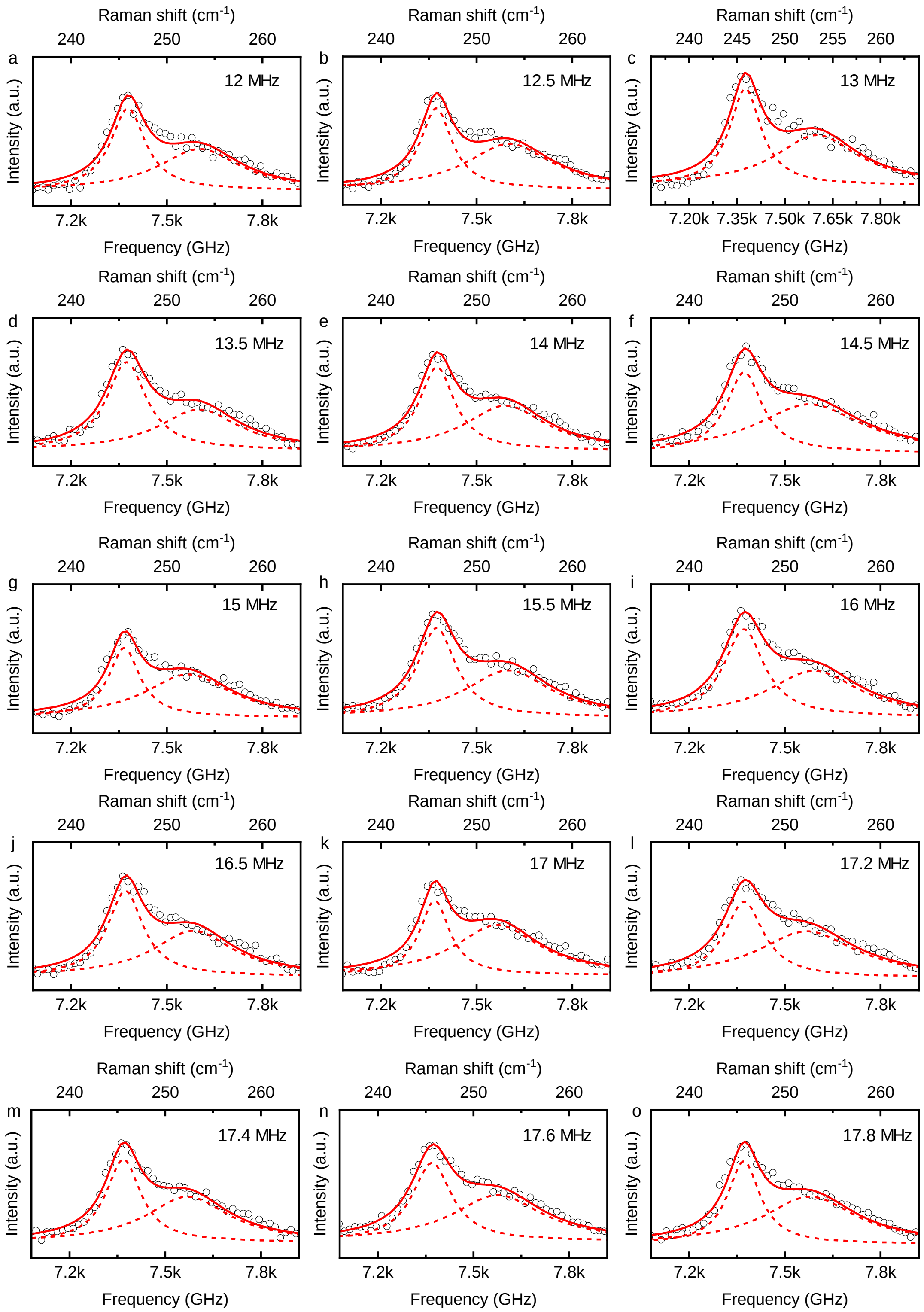

Fig. S3 Fitting of Raman spectra for the 4L WSe_2 device at different driving frequencies (12-25 MHz) with fixed driving amplitude v_{ac} = 800 mV and DC bias V_g = 7 V. Symbols are measured spectra data and dashed lines are fittings to the individual peaks, with solid lines being the sums.

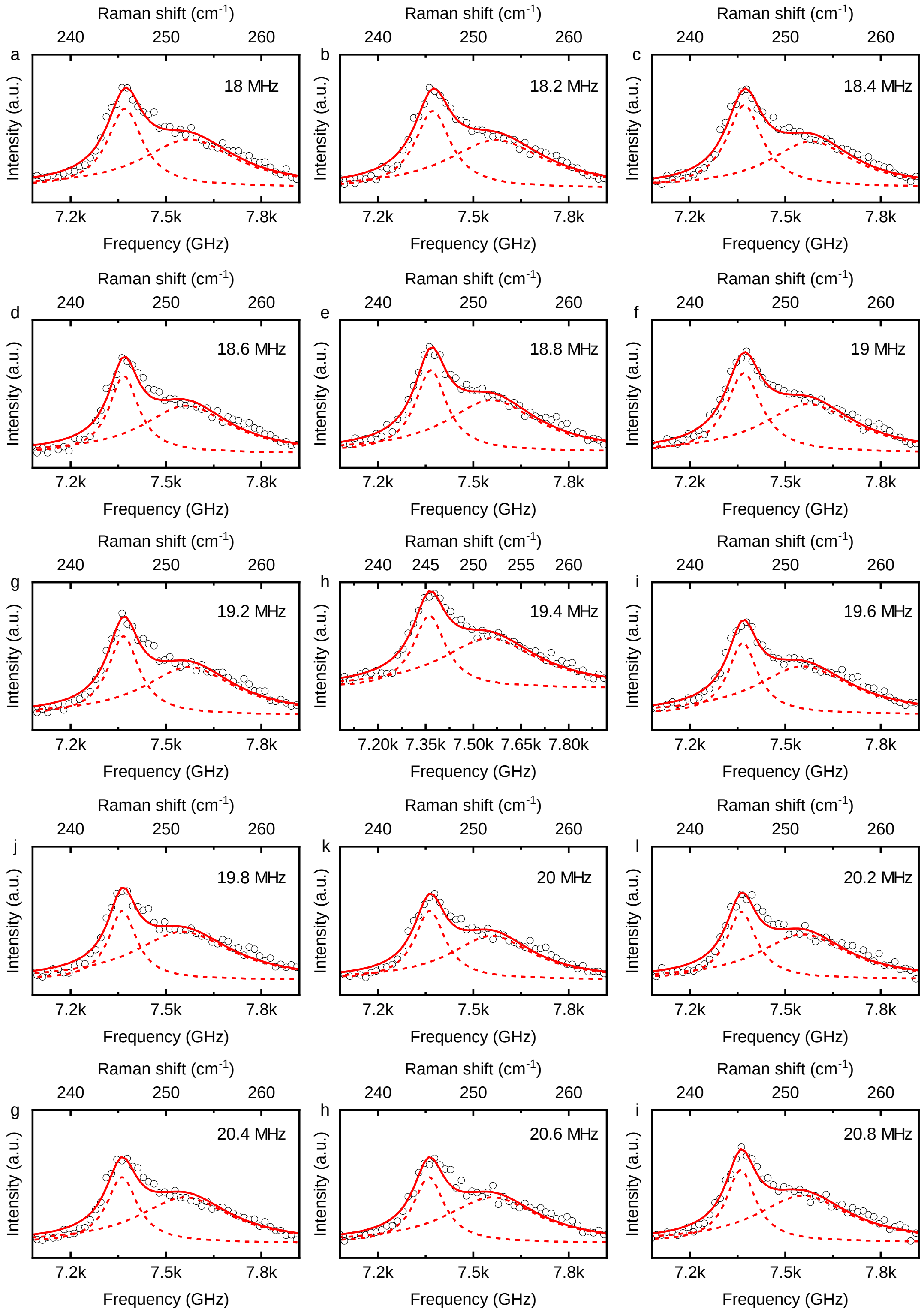

Fig. S3 (Continued from previous page).

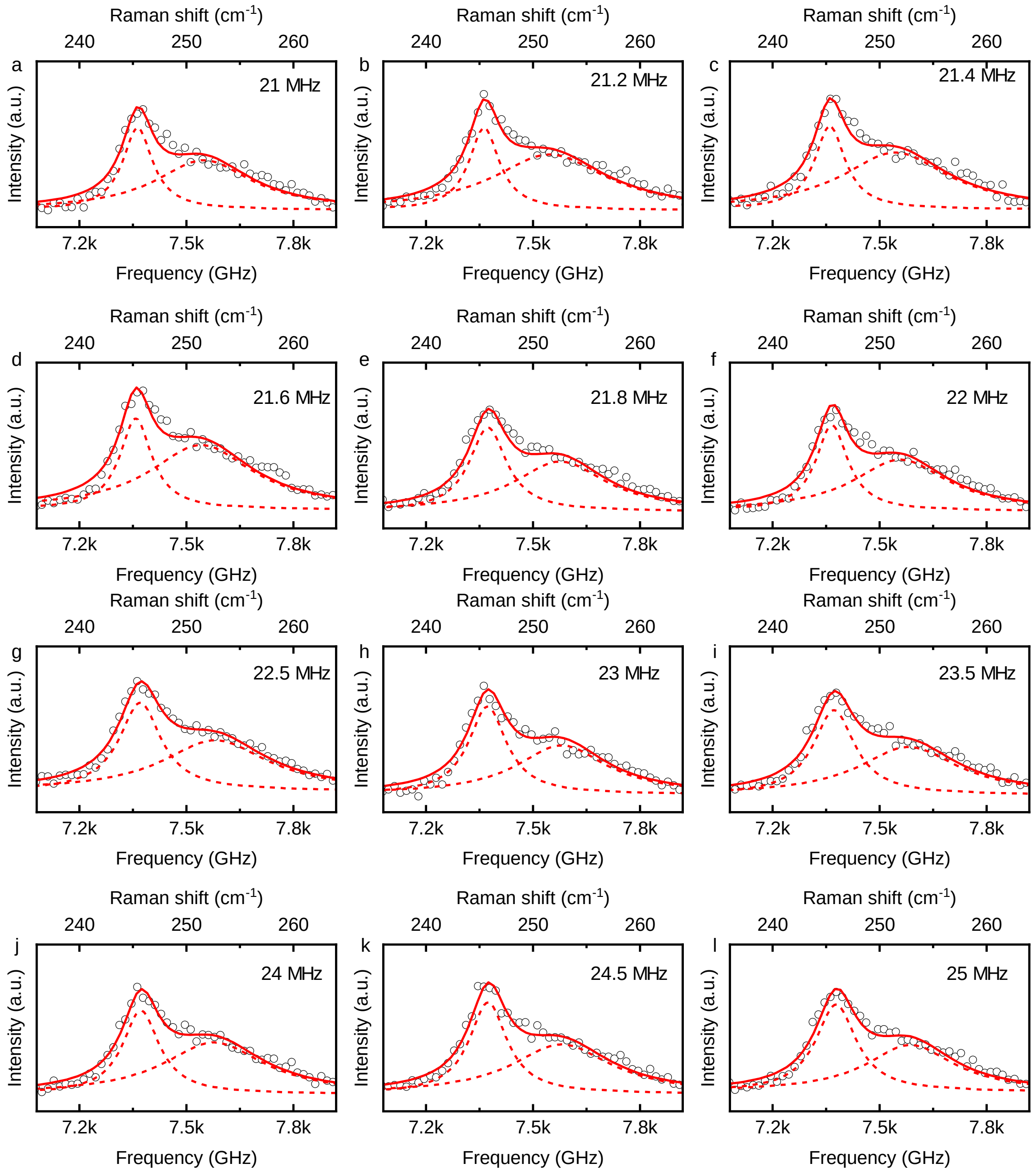

Fig. S3 (Continued from previous page).

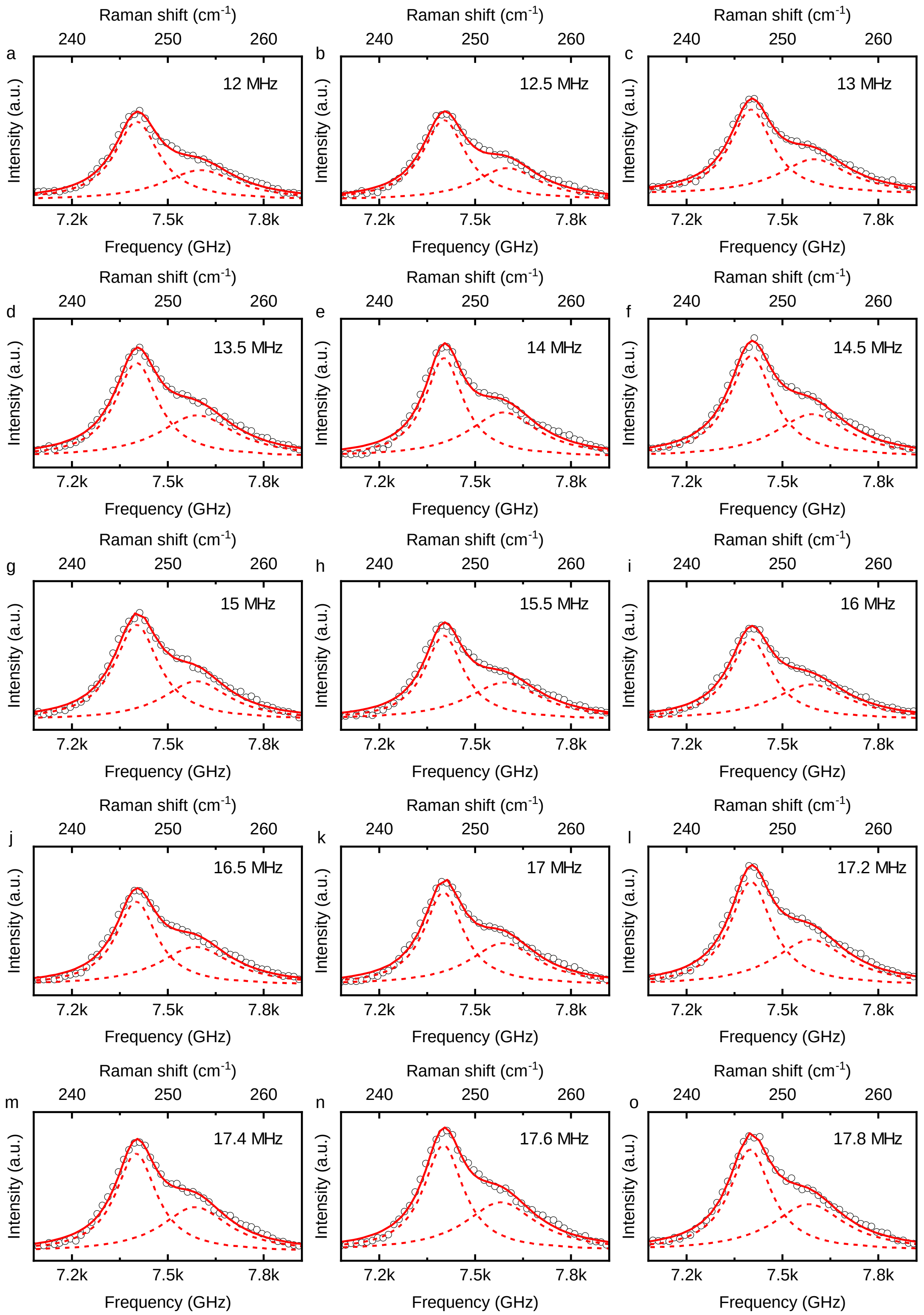

Fig. S4 Fitting of Raman spectra for the 3L WSe_2 device at different driving frequencies (12-22 MHz) with fixed driving amplitude v_{ac} = 200 mV and DC bias V_g = 6 V. Symbols are measured spectra data and dashed lines are fittings to the individual peaks, with solid lines being the sums.

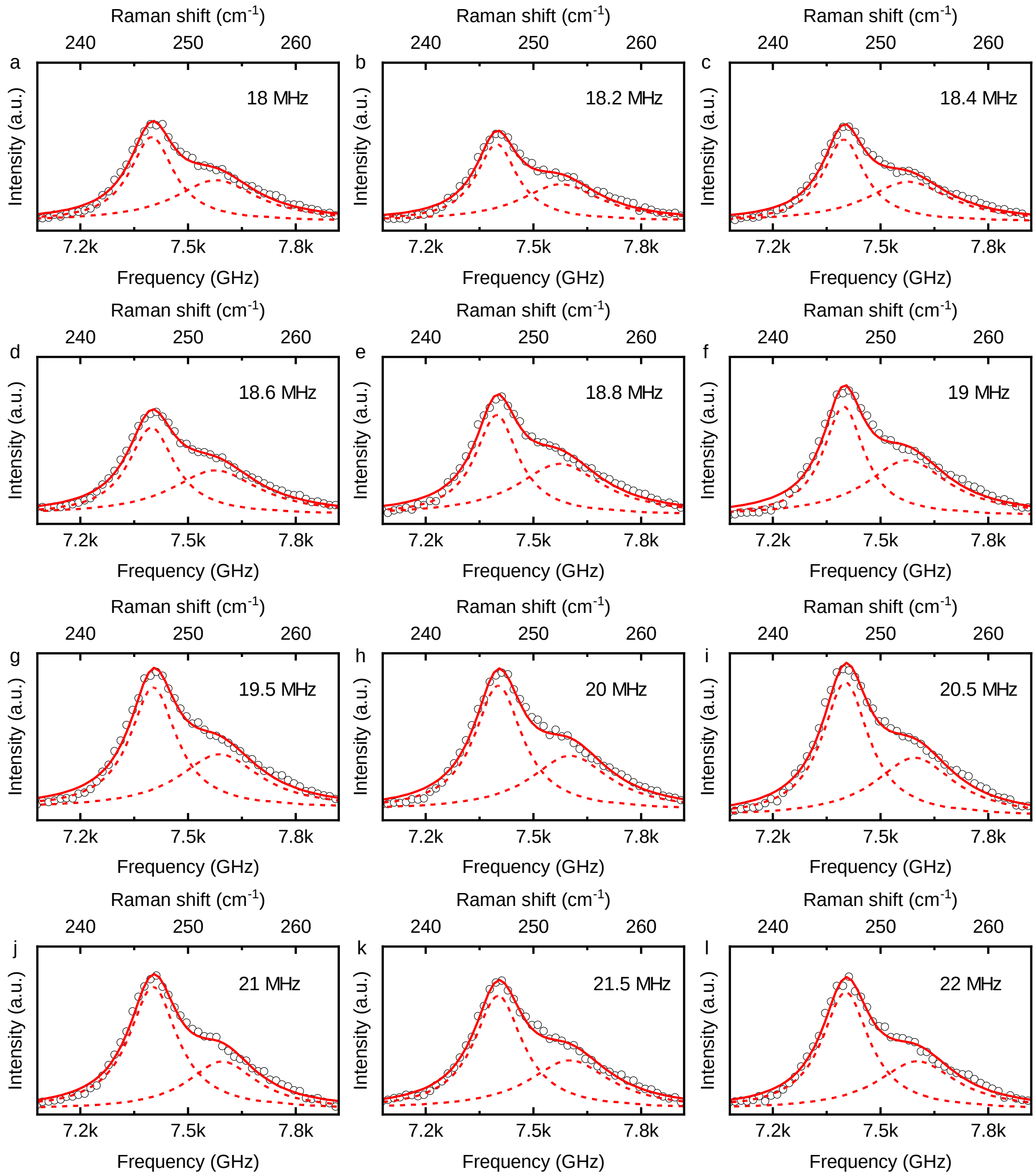

Fig. S4 (Continued from previous page).

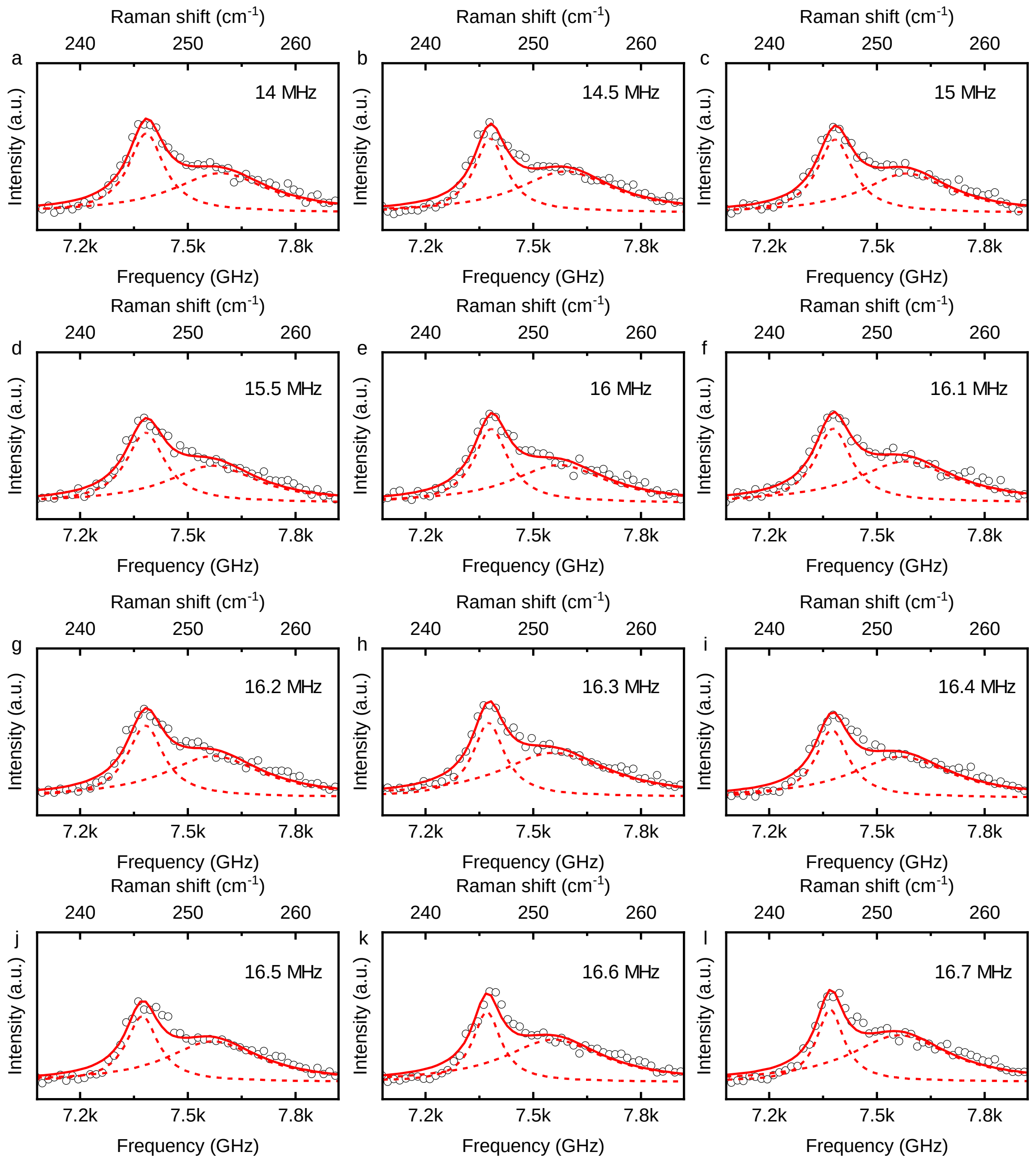

Fig. S5 Fitting of Raman spectra for the 5L WSe_2 device at different driving frequencies (14-18 MHz) with fixed driving amplitude v_{ac} = 60 mV and DC bias V_g = 6 V. Symbols are measured spectra data and dashed lines are fittings to the individual peaks, with solid lines being the sums.

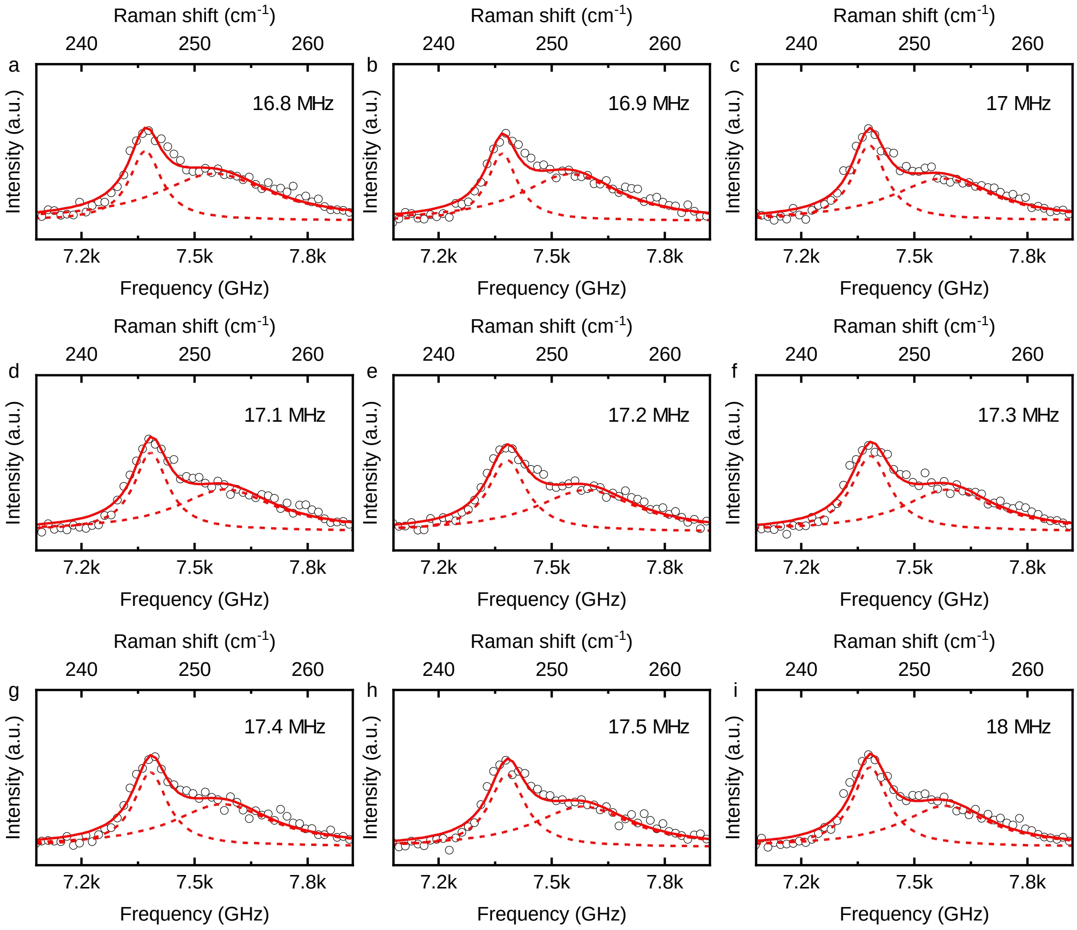

Fig. S5 (Continued from previous page).

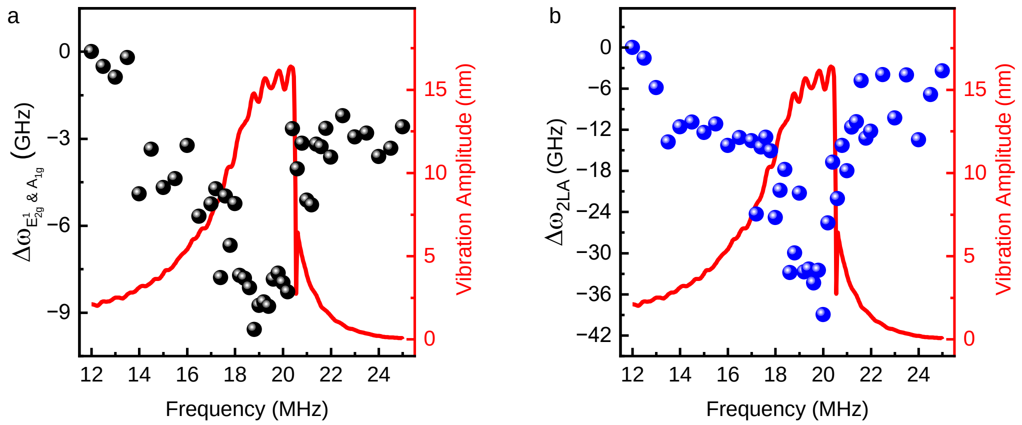

Fig. S6 Measured nonlinear resonance spectrum during downward frequency sweeps (red line, right axis), and Raman peak shift at different driving frequencies for E_{2g}^{1} & A_{1g} (a) and 2LA (b) modes (black and blue symbols, left axis).

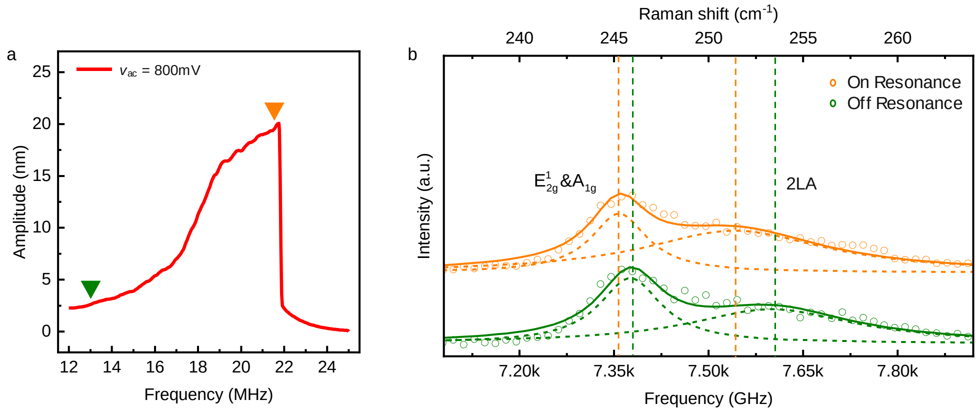

Fig. S7 Resonances and Raman spectra measured from the 4L WSe_2 NEMS resonator in Fig.2. (a) Resonance spectra measured with DC gate voltage V_g = 7 V, RF voltage v_{ac} = 800 mV. (b) Raman spectra of the device driven at V_g = 7 V, v_{ac} = 800 mV, showing E_{2g}^1 & A_{1g} and 2LA modes, with the driving frequency off resonance (green) and on resonance (orange). The measurement frequencies are indicated by the triangles in (a).

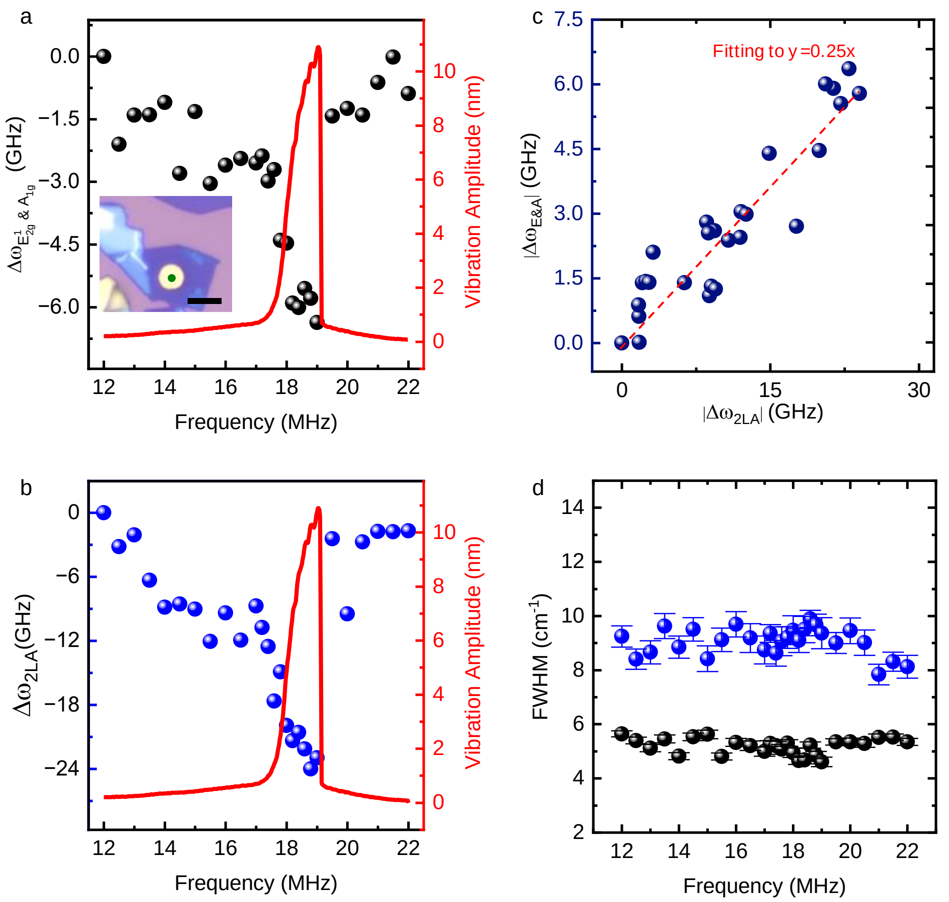

Fig. S8 Resonance and Raman spectra measured from the 3L WSe_2 (d = 4 μm) device (inset in a; scale bar: 5 μm; in which the green dot indicates the laser spot position) with V_g = 6 V and v_{ac} = 200 mV. (a)&(b) Measured nonlinear resonance spectrum (red, right axis) and Raman peak shift. (a) $\Delta\omega_{E2g1\ \&\ A1g}$ and (b) $\Delta\omega_{2LA}$ under different driving frequencies (black and blue symbols, left axis) plotted alongside the resonance response curves. (c) The extracted relationship between E_{2g}^{1} & A_{1g} peak shift ($\Delta\omega_{E2g1\ \&\ A1g}$) and 2LA ($\Delta\omega_{2LA}$). (d) FWHM for E_{2g}^{1} & A_{1g} and 2LA Raman peaks under different driving frequencies. Fitting details of Raman spectra at different driving frequencies are shown in Fig. S4.

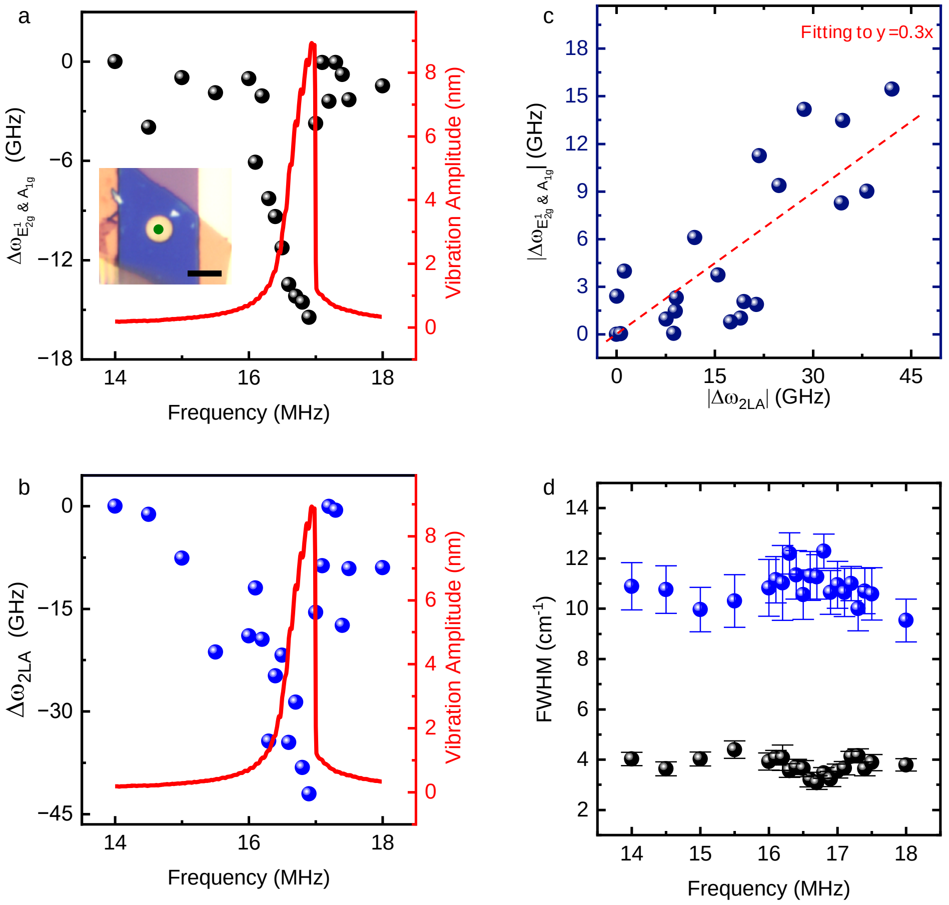

Fig. S9 Resonance and Raman spectra measured from the 5L WSe_2 (d = 4 μm) device (inset in a; scale bar: 5 μm; in which the green dot indicates the laser spot position) with V_g = 6 V and v_{ac} = 60 mV. (a)&(b) Measured nonlinear resonance spectrum (red, right axis) and Raman peak shift. (a) $\Delta\omega_{E2g1\ \&\ A1g}$ and (b) $\Delta\omega_{2LA}$ under different driving frequencies (black and blue symbols, left axis) plotted alongside the resonance response curves. (c) The extracted relationship between E_{2g}^1 & A_{1g} peak shift ($\Delta\omega_{E2g1\ \&\ A1g}$) and 2LA ($\Delta\omega_{2LA}$). (d) FWHM for E_{2g}^1 & A_{1g} and 2LA Raman peaks under different driving frequencies. Fitting details of Raman spectra at different driving frequencies are shown in Fig. S5.

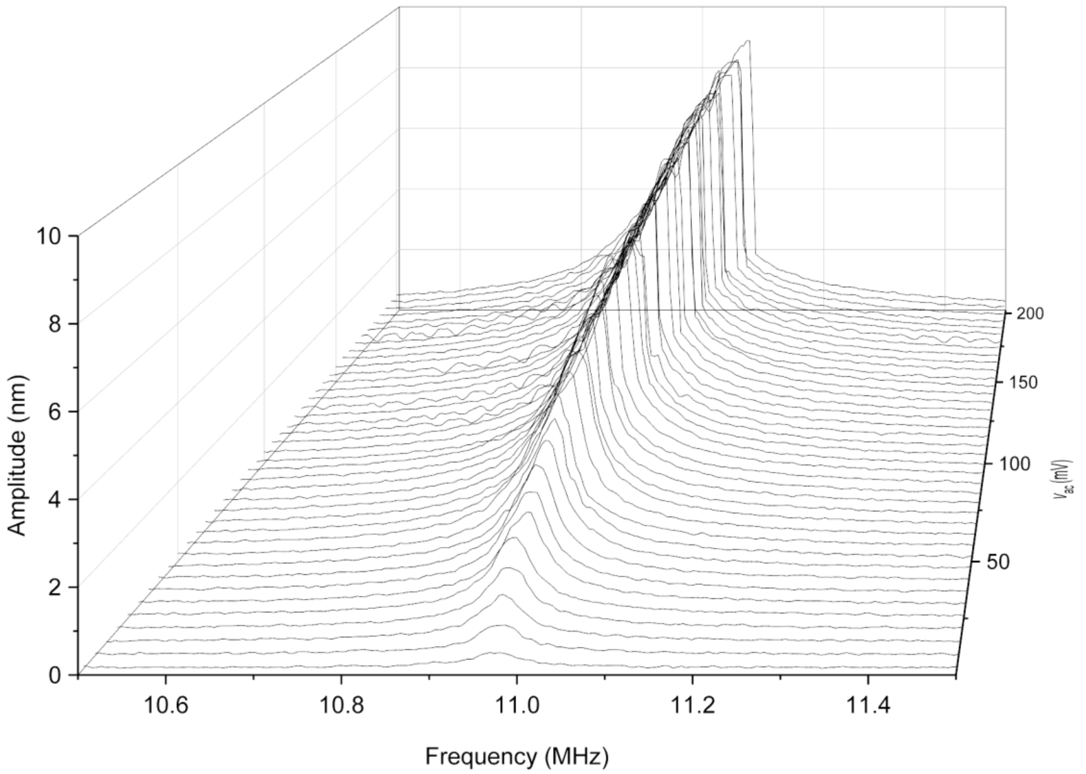

Fig. S10 Nanomechanical vibrations measured from the 3L WSe_2 resonator with d = 4 μm, using V_g = 3 V, and v_{ac} from 5 mV to 200 mV.

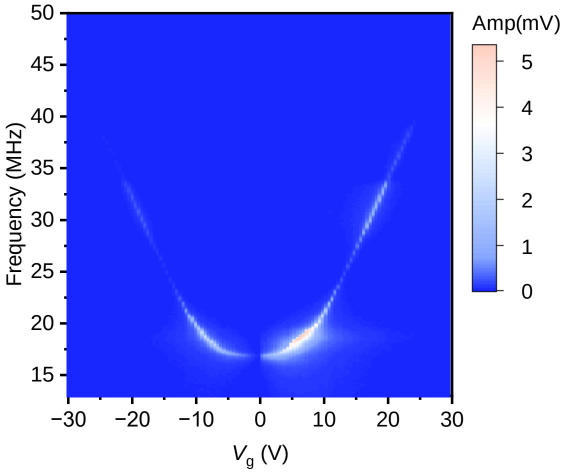

Fig. S11 Color plot of the resonance amplitude of the 4L circular drumhead WSe_2 device with different V_g and with v_{ac} fixed at 30 mV.
